# Preeclampsia management modalities and perinatal death: a retrospective study in Woldia general hospital

**DOI:** 10.1186/s12884-020-02909-9

**Published:** 2020-04-09

**Authors:** Kenean Getaneh Tlaye, Melese Linger Endalfer, Mesfin Wudu Kassaw, Mussie Mulugeta Gebremedhin, Yared Asmare Aynalem

**Affiliations:** 1 College of Health Science, Woldia University, Woldia, Ethiopia; 2 College of Health Science, Axum University, Axum, Ethiopia; 3 College of Health Science, Debre Berhan University, Debre Berhan, Ethiopia

**Keywords:** Preeclampsia, Perinatal death, Management modalities, Woldia

## Abstract

**Background:**

Hypertensive disorders of pregnancy are among the most common causes of perinatal death. The disorders are highly linked to multiple factors that make prediction and prevention challenging. Early diagnosis and proper management play a crucial role in the wellbeing and life of the women and her baby. In this study, we aimed to assess the association between different management options of preeclampsia and perinatal death at a public hospital in Ethiopia.

**Methods:**

A document review was conducted on 241 preeclamptic patients’ medical files who have been admitted and delivered in Woldia General Hospital from 2011 to 2016. The study was conducted from August 8 – September 10, 2017 in the aforementioned public hospital in Woldia town, Ethiopia. Associations were tested using Pearson chi squared test and binary logistic regression with a *p*-value < 0.05 considered significant.

**Result:**

In this study, nearly 20 every 100 neonates from preeclamptic women has been died and the figure was higher (76.59% Vs 23.4%) among neonates from severe preeclamptic women than mild preeclamptic women (*p* = 0.01). More than two thirds of the patients (69.3%) received magnesium sulfate to prevent convulsion. Perinatal death among women with diastolic blood pressures greater than 110 mmHg at admission was nearly 3 times (Adjusted Odds Ratio (AOR) = 2.824; 95% Confidence Interval (CI) (1.154–6.038)) higher compared to women with diastolic blood pressures below 110 mmHg.

**Conclusion:**

In the 5-year period, the magnitude of perinatal death among inpatient preeclamptic women was remarkably high and of which stillbirths exceeded pre-discharge early neonatal death. Utilization of magnesium sulfate tended to increase across years. Maternal diastolic blood pressure at admission was significantly associated with perinatal death.

## Background

According to the Ethiopian Demographic and Health Survey, perinatal mortality rate is calculated as the sum of stillbirths and early neonatal deaths divided by the total sum of births expressed per 1000 pregnancies that lasted seven or more months [1]. Globally, nearly 16% of the estimated 2.6 million stillbirths annually occur in pregnancies complicated by pregnancy induced hypertension [2]. Hypertensive disorders of pregnancy (HDP) precede 10% of early neonatal deaths (8/1000 live births) [3] and a significant proportion of late neonatal deaths (3/1000 live births) [4]. Even though HDP by itself impact the fetus and its survival after delivery, the management modalities used have also been associated with perinatal death.

Studies have shown that nationwide preeclampsia-related perinatal mortality was highly linked with the country’s economic index i.e., figures from high wealth index countries were low and disproportionally high perinatal mortality were reported from low- and middle-income countries (LMIC). In general, rates ranged from 4.7% to as high as 41.6% [5–8]. Similarly, perinatal mortality among patients with eclampsia was also reported to be 5 to 11% in High income countries (HIC) where as it was as high as 40% in LMIC [9, 10]. Two studies conducted in public hospitals in Ethiopia; namely Jimma University Specialized Hospital and Mettu Karl Referral Hospital; revealed perinatal mortality among preeclamptic women were 317/1000 births and 120/1000 respectively [11, 12].

A study showed that inadequate and delayed initiation of treatment and preterm deliveries among preeclamptic women was found to be associated with poor fetal outcome [13]. As variation is perceived among the course of management modalities used by different institutions, its impact on perinatal death should be explored especially in resource limited areas. Therefore, the aim of this study was to assess the perinatal death rate and its association between preeclampsia management modalities and perinatal outcome among women who gave birth at Woldia General Hospital from 2011 to 2016.

## Methods

### Study setting, design and eligibility criteria

**Fig. 1:**
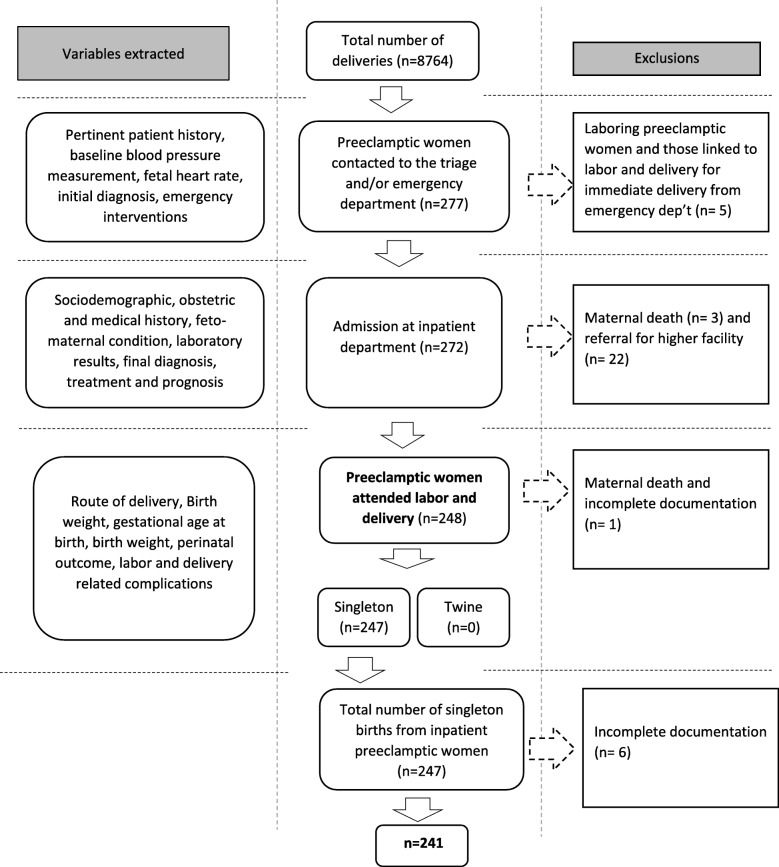
Flow chart illustrating the sampling procedure, variables extracted at each department and cases excluded from the study on preeclampsia management modalities and perinatal death at Woldia General Hospital

The study was conducted from August 8 – September 10, 2017 at Woldia General Hospital, Woldia, Ethiopia. Woldia town is the main town of North Wollo Zone. This town is located in Amhara region at 520 km North of the capital, Addis Ababa. According to the local administration report in 2016, the town has total population of 75,446 of which 38,167 were males and 37,279 were females. Regarding the health care system, the town has one public hospital, two public health center and 12 private pharmacies and 5 medium level clinics. The hospital provides both inpatient and outpatient services. At the time of the study, the hospital had 120 beds and 328 employees and of them there were 20 doctors, 128 nurses and 21 midwifes. It has an obstetrics and gynecology department but until the data collection time, there wasn’t separate room to admit patients with preeclampsia. A hospital-based retrospective cross-sectional study was implemented. All preeclamptic women who were admitted and gave birth at Woldia General Hospital from September 1, 2011- September 1, 2016 were included for the review. Perinatal outcome which was dichotomized as alive or dead was assessed for neonates delivered after 28 completed weeks and through their early neonatal period before discharge (≤ 7 days). As the study was planned to assess the effect of different patterns of preeclampsia management on perinatal death, subjects’ medical files documented with coincidental appearance for possible causes of perinatal death were excluded. This included maternal death on arrival, during admission and labor, and multiple delivery. In addition, grossly incomplete medical files; subjects with the initial diagnosis of preeclampsia in the first-time at the emergency department and immediately delivered were also excluded. The reason for the latter was the author’s assumption that much of the inpatient management scheme that of interest for the present study would have missed and there might also be the probability of misdiagnosis. Figure 1 showed the schematic representation of sampling procedure and excluded cases from each departments *(*Fig. 1*).*

**Table 1:** Obstetric and medical condition of patients at the time of admission

Obstetric and medical condition at Admission	mean (SD)
Gestational age during admission (in weeks ^days^) (*n* = 239)	37^+ 6^ (±2^+ 5^)
Diastolic blood pressure at admission (mmHg)	105.87 (±10.58)
Obstetric and medical condition at Admission	**n (%)**
Gravidity	
Primigravida	138 (57.3%)
multigravida	103 (42.7%)
Antenatal follow-up for current pregnancy	
Yes	168 (69.7%)
No	73(30.3%)
Comorbidities^a^(*n* = 98)	
Yes	57 (58.2%)
No	41 (41.8%)
Dipstick proteinuria (*n* = 223)	
Mild proteinuria	148 (66.4%)
Marked proteinuria	75 (33.6%)
Degree/type of preeclampsia diagnosed	
Mild preeclampsia	99 (41.1%)
Severe preeclampsia	139 (57.7%)
Preeclampsia superimposed on chronic hypertension	3 (1.2%)
Sign and symptom of end organ involvement	
Headache (*n* = 228)	
Yes	111 (48.7%)
No	117 (51.3%)
Visual problem (*n* = 229)	
Yes	30 (13.1%)
No	199 (86.9%)
Epigastric pain (*n* = 213)	
Yes	18 (8.5%)
No	195 (91.5%)
Abruptio placenta (*n* = 117)	
Yes	
No	117 (100%)
Thrombocytopenia (*n* = 58)	
Yes	3 (5.2%)
No	55 (94.8%)
Elevated liver enzymes (*n* = 52)	
Yes	
No	52 (100%)

^a^assessed only for HIV, diabetes, chronic hypertension and anemia

**Table 2:** Time duration (in hours) for the patients and health care professionals to start some management measures on preeclamptic women admitted at Woldia General Hospital

Time lapses	median (interquartile range) in hours
Time lapsed from onset of current symptoms to reach hospital (*n* = 236)	14 (6.25–24)
Time lapsed from admission to beginning of MgSO4^a^ (*n* = 139)	1 (0.5–1)
Time lapsed from admission to beginning of antihypertensive medication^b^ (*n* = 101)	0.5 (0.28–1)
Time lapsed from admission to onset of labor^c^ (*n* = 141)	24 (10.25–48)

^a^For patients with severe preeclampsia and preeclampsia superimposed on chronic hypertension

^b^For patients with DBP ≥ 110 mmHg

^c^For women with severe preeclampsia and preeclampsia superimposed on chronic hypertension

### Data collection tools and quality assurance

The data were collected using a pre-tested check list which assessed socio-demographic variables, obstetrics history, signs and symptoms at presentation, laboratory results, and perinatal outcomes. The data were obtained from admission registration logbooks, medical and nursing care progress notes, delivery registration books, laboratory and drug prescriptions. The data collectors were trained a 2-days intensive training on how to review and extract the required information from the patients’ chart. They were also monitored by supervisors daily. The tool was pretested at Lalibela General Hospital and an effort was made to validate patients’ data from different folders and registration books.

### Study variables

The study outcome variable was a perinatal outcome from preeclamptic women, which was dichotomized as alive and dead. The independent variables were, sociodemographic factors such as patients age and residence; obstetric related factors; gestational age (at admission and delivery), ante-natal follow up for current pregnancy; current medical history (comorbidities); conditions at admission such as blood pressure measurement, level of proteinuria and signs and symptoms for end organ involvement, treatment modalities including; anti-hypertensive modalities, anti-convulsion modalities, mode of delivery; and other perinatal related factors such as intra-partum fetal heartbeat, APGAR score and birth weight.

### Operational and term definitions

A patient medical file was documented as incomplete if it failed to mention perinatal outcome (as dead or alive), or if greater than 20% of the independent variables were missed. In this study, perinatal death included stillbirths (fetal death inside the uterus after 28 weeks) and early neonatal deaths (deaths within the first week after delivery). Gestational ages reported in the patient’s file was considered valid if it was calculated from the last normal menstrual period or using obstetric ultrasound. Fetal heart beat was usually obtained with Pinard fetoscope and a presence of any single value lower 100 bpm or above 160 bpm, classified as abnormal pattern. We have used the routine clinical classification of preeclampsia as mild and severe. Hence, cases of documented blood pressure of more than 140/90 mmHg but less than 160 mmHg systolic or 110 mmHg diastolic with qualitative dipstick proteinuria less than + 2 (mild proteinuria) was labeled as mild whereas a persistent systolic blood pressure of > 160 mmHg or diastolic pressure of > 110 mmHg with significant protein excretion of greater than + 3 (marked proteinuria) or suggestive evidence for end organ involvement was recorded as severe preeclampsia. If preeclamptic women reported at least one of these symptoms; headache, visual problem, epigastric pain and placental abruption, platelet count below 100,000/mm^3,^ or aspartate aminotransaminase (AST) > 70 IU/L, the subject was labeled as having signs of end-organ involvement. Comorbidity status for HIV, diabetes mellitus, chronic hypertension and anemia was assessed as either ‘Yes/No’ based on patient’s self-report or laboratory result. Labor and early neonatal complications were assessed by a positive finding for at least one of the following complications: meconium aspiration, cord prolapse, vasa previa, prolonged labor (more than 24 h.), uterine rupture, neonatal tetanus, severe jaundice, neonatal sepsis, life threatening congenital defect. Women with hemoglobin level less than 11 g/dl was reported as anemic and classified as severe (Hgb < 7.0 g/dl) moderate (Hgb 7.0–9.9 g/dl) and mild (Hgb 10.0–10.9 g/dl). Time-lapse measurements were taken in hours and categorized based on their mean, median, or percentile after checking for their normality.

### Data processing and analysis

Data were entered and analyzed by Statistical Package for the Social Science (SPSS) version 23 (RRID:SCR_002865). Percentage, rate, frequency distribution, measure of central tendency such as mean and median as well as measure of dispersion like interquartile range and standard deviation were used to describe different variables. Continuous variables such as time measurements were checked for their normality and reported using mean and standard deviation if normally distributed or median and interquartile range (IQR) if skewed. Pearson’s χ2 test were used to examine association between selected variables. The effect of each variable on perinatal death was first tested by bivariable logistic regression. Confounding bias imposed by independent variables was further controlled by multivariable logistic regression. Association was considered significant when the *p*-value was less than 0.05 and adjusted odds ratio values were reported along with their 95% confidence interval. For a better calculation of the effect of preeclampsia and its management schemes on perinatal death, the study tried to assess the effect of other possible causes of perinatal death using the variable ‘labor and early neonatal complication’ and controlled with multivariable logistic regression.

## Result

There were 8764 deliveries attended at Woldia General Hospital in the study period of which 248 were diagnosed as preeclamptic and gave birth in the hospital. Seven medical files were further excluded due to maternal death and incomplete documentation and therefore findings from this study were reported based on 241 reviewed medical documents. The mean maternal age in years (±SD) was 25.73 ± 4.8 with the minimum of 15 years and maximum of 40 years. One hundred thirty-one (54.4%) of them were rural dwellers (Table 1).

The study also assessed labor and early neonatal period complications that leads to possible neonatal death. According to the review, 20 of such complications were documented of which half were meconium aspiration.

### Patterns of preeclampsia management modalities

The median time lapsed for the patients from onset of their symptoms to arrive to the hospital was 14 h. A wide variability has been observed on the duration of time from admission to the onset of labor among patients diagnosed with severe preeclamptic and preeclampsia superimposed on chronic hypertension. Thus, the mean (±SD) hour was 44.9 (±39.1) with the minimum of 1 and maximum 192 h (8 days) duration (Table 2).

**Fig. 2:**
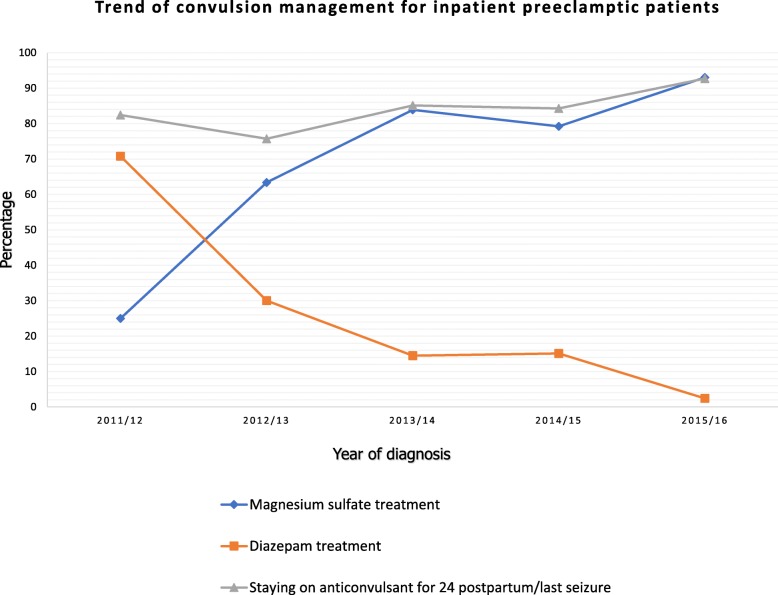
Trend of convulsion management modalities among inpatient preeclamptic women admitted at Woldia General Hospital from 2011 to 2016

Magnesium sulfate was the most frequently (69.3%) used anticonvulsant medication used followed by diazepam (26.6%). The most frequently used antihypertensive medications were hydralazine, methyldopa and nifedipine that were used 112, 111 and 54 times respectively. Anticonvulsant medication was given for 221 (91.7%) during labor and its use continued for 24 h postpartum or since last convulsion for 177 (73.4%) patients. Out of 239 patients, 148 (61.9%) has commenced their labor spontaneously while it was intentionally induced for 91 (38.1%) patients. Spontaneous vaginal delivery was the major route of delivery 114 (47.5%) while 82(34.2%), 35(14.6%), and 9(3.75%) of patients managed by vacuum, caesarian section and forceps delivery respectively. Comparing the route of delivery with severity of the disease (classifid as mild or severe preeclampsia), the use of vacuum delivery and caesarean section were at a statistically higher frequency among severe preeclamptic women over their counter parts x^2^(3, *n* = 117) = 2.398, *p* = 0.013, while no significant differences were observed in the frequency of spontaneous vaginal delivery (*p* = 0.613) (Fig. 2)*.*

**Table 3:** A bivariable and multivariable logistic regression analysis result testing for factors associated with perinatal outcome (death/alive) among neonates delivered from preeclamptic women from 2011 to 2016 (before the effect of labor and early neonatal complication controlled)

Variable		COR, (95% CI)	***p***-value	AOR, (95% CI)	***p*** value
Diastolic Blood pressure measurement (*n* = 231)	Less than 110 mmHg	1	0.009	1	0.335
	≥ 110 mmHg	3.688(1.257–4.863)		1.606(0.034–3.177)	
Manifestation of end-organ involvement	Yes	4.667 (1.332–8.001)	0.012	4.404 (1.219–6.370)	0.035*
	No	1		1	
Gestational age at birth	< 37 completed weeks	2.547 (1.073–4.021)	0.030	1.105 (0.098–2.111)	0.315
	≥ 37 completed weeks	1		1	
Time lapsed from admission to initiation of MgSO_4_^b^ (*n* = 139)	Less than 1 h	1		1	
	More than 1 h	1.254 (0.343–2.164)	0.751	2.780 (0.558–5.002)	0.427
Time lapsed from admission to initiation of antihypertensives^c^ (*n* = 101)	Less than 17 min	2.962 (0.331–5.593)	0.669	3.636 (0.231–7.014)	0.125
	17–30 min	3.436 (0.479–6.392)	0.397		
	30 min to 1 h	2.331 (0.195–4.467)	0.931		
	More than or equal to 1 h	1		1	
Time lapsed from admission to onset of labor^d^ (*n* = 141)	Less than 35 h	1		1	0.412
	More than or equal to 35 h	1.353 (0.235–2.452)	0.644	1.951 (0.896–3.006)	
Qualitative proteinuria (*n* = 223)	Mild proteinuria	1	0.776	1	0.623
	Marked proteinuria	1.395 (0.547–2.243)		1.159 (0.113–2.091)	
MgSO_4_ continues 24 h after delivery (*n* = 210)	Yes	1	0.024	1	
	No	3.132 (1.048–4.168)		4.069 (0.896–6.347)	0.221
Intrapartum Fetal heart rate (*n* = 225)	Normal	1	0.014	1	0.112
	Some abnormal records	6.328 (2.069–10.587)		4.846 (0.667–9.025)	
Onset of labor	Spontaneous	1		1	0.119
	Induced	5.580 (2.237–8.923)		7.477 (0.633–14.322)	
Labor and early neonatal complications (*n* = 239)	Yes	3.454 (1.321–5.588)	0.006	4.616 (1.365–7.867)	0.017*
	No	1		1	
Fifth minute APGAR score^a^ (*n* = 214)	0–3	13.962 (6.499–21.426)	0.011	5.046 (1.856–8.235)	0.0005*
	4–6	7.645 (2.629–12.660)	0.003	3.112 (1.223–5.001)	0.021*
	7–10	1		1	

Key: COR: Cruds odd ratio

*show association with p value less than 0.05

^a^analysis was done after excluding stillbirths

^b^For patients with severe preeclampsia and preeclampsia superimposed on chronic hypertension

^c^For patients with DBP > 110 mmHg

^d^For women with severe preeclampsia and preeclampsia superimposed on chronic hypertension

As shown in the Fig. 2, magnesium sulfate was the drug of choice to prevent convulsion in preeclamptic women. Its use increased from 25% in 2011/12 to 93% in 2015/16 (*P* < 0.0005). Opposite to this, there was a decreasing trend for using diazepam from 54% in 2011/12 to 1.6% in 2015/16 (*P* < 0.0005). In addition, extending the use of anticonvulsant medication 24 h postpartum or after the onset of last convulsion increased across the 5 years.

### Perinatal outcome

**Fig. 3:**
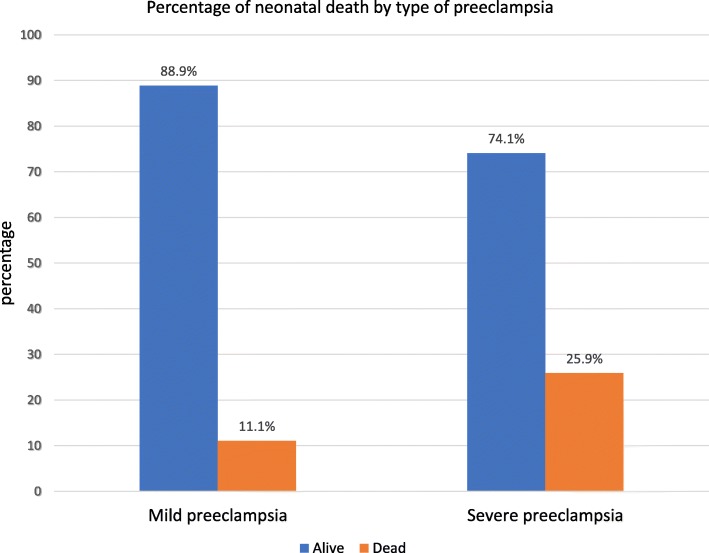
Percentage of perinatal death versus type preeclampsia at Woldia General Hospital from 2011 to 2016

The mean gestational age in weeks and days at birth was 37^+ 6^ (±2^+ 5^) and the minimum and the maximum value of 24 and 42 completed weeks respectively. The mean value for 1st and 5th minute APGAR score was 5.98 (±2.36) and 6.91 (±2.67) respectively. The mean value for birth weight was 2608.02 (± 558.8) gram. Low birth weight was more frequently observed among severe preeclampsia than the mild one (53.7% Vs 32%) *p* = 0.001. Out of 225 documents reviewed for fetal heart beat abnormalities, 24 (10.7%) abnormal patterns were documented. Forty-Seven perinatal deaths were recorded across the 5-year period yielding a case specific perinatal mortality rate 197/1000 births. Of this, 27(57.4%) were documented as stillbirth and the rest 20 (42.6%) were within the first week of their hospital stay. Perinatal death was higher (76.59% Vs 23.4%) among offspring from severe preeclampsia patients than their mild counterparts (*p* = 0.01) *(*Fig. 3*).*

Using logistic regression, important independent variables were checked for their possible association both in bivariable and multivariable logistic regression with a *p*-value less than 0.05. Three variables were significantly associated with perinatal death both in bivariable and multivariable logistic regression; namely intrapartum fetal heart rate pattern, manifestations of end organ involvement, and 5th minute APGAR score. It was observed that the odds of perinatal death among those with manifestation of end-organ involvements were 4 times (AOR = 4.404 95% CI (1.219–6.370)) higher than their counterparts (Table 3).

We found that a group of conditions operationalized as ‘labor and early neonatal complications’ increased the odds of perinatal death nearly by 5-fold. However, this variable is an independent cause of perinatal death even in the absence of preeclampsia. Therefore, the multivariable logistic regression was repeated in which ‘labor and early neonatal complications’ and other independent variables were entered into the regression model with separate blocks. From the final analysis, it was found that the diastolic blood pressure measurement and 5th minute APGAR score were associated with perinatal death. Accordingly, the odds of perinatal death was nearly 3-times (AOR = 2.824; 95%CI (1.154–6.038)) higher among women who had diastolic blood pressures greater than 110 mmHg taken at admission compared to those who had below 110 mmHg. Neonates whose 5-min APGAR score was between 0 to 3 has a 5-fold (AOR = 5.046; 95% CI (1.856–8.235)) greater risk of death than neonates whose 5-min APGAR was between 7 and 10*.*

## Discussion

The time-lapsed from onset of symptoms to the patient reaching health care support is a combined reflection of patient’s health-care seeking behavior, access to health care, infrastructure and transportation. Regarding the management modalities used, we believed that in addition to comparing this study finding with others’, discussing our findings with national guideline and protocols will help to identify the magnitude of management gap.

In previous research, the rate of perinatal mortality among those mothers with preeclampsia ranged from 47/1000 to 416/1000 live births [5–8]. The perinatal mortality rate recorded in our study (197/1000 live births) was within this range but slightly higher than two studies with rates of 120/1000 and 160/1000 deaths per live births [2, 12]. Differences in death rates might arise from difference in the gestational age cut off points used among studies, the type of hypertensive disorders included, and service-delivery capacity of the hospitals where the patients were managed.

Similar to other studies, in this sample, 47.5% of all preeclamptic women had vaginal births [14, 15]. Vaginal delivery is also the preferred route of delivery for preeclamptic women according to the Ethiopian Minister of Health protocol [16]. Rates of labor induction vary a great deal across studies. Similar to a rural hospital in Western Tanzania, in this study labor was induced in 38.1% of the patients [17]. In contrast, 47.9% of patients were induced at the Mettu Karl Referral Hospital [12] and 23.4% in governmental hospitals of Addis Ababa [14]. The possible explanation for this difference could be the fact that labor induction has a variety of chief indications other than preeclampsia.

In three studies, usage of magnesium sulfate in preeclamptic women to prevent seizures ranged from 74.1 to 100% [12, 14, 17] similar to our finding of (91.7%). In addition to this, this study revealed that anti-convulsant medication was continued for 24 h postpartum for 177 (73.4%) patients. Although this finding was consistent with the aforementioned studies conducted in resource-limited areas, it was lagged behind to achieve the Ethiopian Ministry of Health management protocol which recommends the use of magnesium sulfate for all preeclamptic woman during labor. It’s also recommended that magnesium sulfate treatment should continue for 24 h postpartum [16, 18]. Unavailability of the drug or failure either to give the drug or to document could be the possible explanation for this discrepancy.

The median duration for women with severe preeclampsia between admission and labor was 24 h (IQR 10.25–48) which was shorter than other study conducted among patients with severe preeclampsia and eclampsia [17]. Shorter hospital stay for severe preeclamptic women at term was stated as a supported management option in studies and management protocols [16, 19].

Similar to our finding, other studies in low-resource settings reported more stillbirths than deaths at early neonatal period [15, 20, 21]. We found that women who had diastolic blood pressures above 110 mmHg had a 2-fold increased chance of perinatal death compared to those with lower diastolic blood pressures. Similar association has been also detected in other studies [22, 23].

### Limitation

As a limitation, this study didn’t assess the effect of steroid drugs on perinatal death among preeclamptic women as only few numbers of cases received the treatment.

## Conclusion

In conclusion, perinatal death was high among inpatient preeclamptic women who gave birth at Woldia General Hospital. The trend of using magnesium sulfate as an anticonvulsant increased through the 5-year period. However, there was a gap to extend its use up to 24 h postpartum or since the last convulsion as per the national protocol. From our analysis, diastolic blood pressure and 5th minute Apgar score were found associated with perinatal death. Authors recommended that future large-scale health service auditing and impact assessment studies in such resource limited-areas might serve as an input for policymakers to improve the quality of clinical care and to decrease mortality rates.

## Data Availability

The datasets used for analysis during the current study are available from the corresponding author on reasonable request.

## Abbreviations

APGAR: Appearance, Pulse, Grimace, Activity, Respiration
HDP: hypertensive disorders of pregnancy
Hgb: Hemoglobin
